# Molecular Analysis of *Staphylococcus epidermidis* Strains Isolated from Community and Hospital Environments in China

**DOI:** 10.1371/journal.pone.0062742

**Published:** 2013-05-13

**Authors:** Xin Du, Yuanjun Zhu, Yan Song, Tianming Li, Tao Luo, Gang Sun, Chongguang Yang, Cuiming Cao, Yuan Lu, Min Li

**Affiliations:** 1 Department of Laboratory Medicine, Huashan Hospital, Shanghai Medical College, Fudan University, Shanghai, China; 2 Key Laboratory of Medical Molecular Virology, Institutes of Biomedical Sciences and Institute of Medical Microbiology, Fudan University, Shanghai, China; 3 Department of Laboratory Medicine, Jinan Central Hospital Affiliated to Shandong University, Jinan, Shandong Province, China; The University of Hong Kong, China

## Abstract

**Background:**

*Staphylococcus epidermidis* is a common cause of nosocomial infections worldwide. This study analyzed the differences in genetic endowment and clonal lineages with pathogenesis and resistance traits of *S. epidermidis* isolates collected from community and hospital environments (patients and healthcare staff) of the same ecological niche, time period, and geographical location in China.

**Methodology/Principal Findings:**

Molecular epidemiology and population analysis showed that nasal colonization rates of *S. epidermidis* in the community of Shanghai area of China and in healthcare personnel were 44.8% (methicillin-resistant *S. epidermidis*, MRSE: 17.2%) and 61.3% (MRSE: 30.0%), respectively. 86.7% of clinical isolates were MRSE. Among the strains studied, 44 sequence types (STs) were identified with 91.7% belonging to clonal complex 2 (CC2). Only 40.8% isolates from patients were also found in healthy individuals. MRSE-ST2-SCC*mec*III was the predominant clone in clinical isolates, almost resistant to all antibiotics tested. Biofilm-related genes *IS256* and *icaA* were detected in majority of the predominant clinical MRSE-ST2 clone with a 40.5% biofilm-positive rate. No ST2 isolate was found in community setting. We found a high prevalence of arginine catabolic mobile element (ACME) (74.1%). The prevalence of ACME-*arc* and ACME-*opp3* clusters was 71.6% and 32.4%, respectively. Methicillin-sensitive *S. epidermidis* (MSSE) isolates harbored more ACME (83.3%) than MRSE isolates (67.7%), and there was no association between ACME and SCC*mec* types. An association was found between low-level ACME presence and invasive infections.

**Conclusions/Significance:**

We observed a high level of diversity within *S. epidermidis* in this study, with CC2 as the dominant clonal complex in both community and hospital settings. Only 40.8% of the isolates from patients were also found in healthy individuals. Contrary to that biofilm formation and multiple antibiotic resistance were associated closely with pathogenicity of *S. epidermidis*, ACME was more likely to be an indicator for colonization rather than a virulence factor.

## Introduction

*Staphylococcus epidermidis* is a commensal bacterium of human skin and the nasal and oral mucosa. However, in recent decades, it has emerged as a common cause of hospital-acquired infections, being responsible for 40–90% of infections associated with indwelling devices in seriously ill or immunocompromised patients [1], [2]. The increasing antibiotic resistance of nosocomial isolates of *S. epidermidis* aggravates this problem and poses a great challenge for the management of hospital-acquired infections in general. Worldwide surveys revealed that 60–85% of clinical strains are resistant to methicillin [3]–[6]. Methicillin resistance is conferred by the *mecA* gene, which is carried by a family of mobile genetic elements called staphylococcal cassette chromosomes (SCCs). To date, 11 types (I–XI) of SCC*mec* have been assigned to *Staphylococcus aureus* based on the class of the *mec* gene complex and the type of *ccr* gene complex present [7], [8]. SCC*mec* has been shown to be transferable among staphylococcal species [9]. Besides SCC*mec*, other SCC elements have been described in staphylococci that transport important survival or virulence genes, such as arginine catabolic mobile element (ACME), a novel genomic island that may contribute to the enhanced capacity of this species to colonize the human skin and mucosal surfaces [10]–[12]. ACME was first identified in the genomic sequence of methicillin-resistant *S. aureus* (MRSA) USA300 in 2006 [10]. It has been assumed that ACME was excised from coagulase-negative staphylococci by *ccr*-encoded recombinase and then transferred horizontally to other staphylococci. However, no data regarding the distribution of the ACME gene among *S. epidermidis* strains in China have been reported.

Molecular typing of nosocomial *S. epidermidis* strains using several methods has shown considerable diversity within the *S. epidermidis* population [13]–[15]. By using multilocus sequence typing (MLST), it was shown that the population structure of *S. epidermidis* in hospital environments worldwide is composed of a major and highly diverse genetic lineage, i.e., clonal complex 2 (CC2) [3], [16], [17]. To our knowledge, a wide proportion of studies on *S. epidermidis* has been conducted exclusively on clinical strains, and much less is known regarding the epidemiology of community-associated *S. epidermidis*. Only a few studies have described the frequency of nasal colonization with MRSE as 20% among healthy Japanese children [18] and 7% among healthy draftees in the USA [19]. Little information is available on the molecular epidemiology of *S. epidermidis* in the community strains circulating in China. Furthermore, medical and healthcare workers are reportedly reservoirs of *S. epidermidis* that can infect susceptible patients [20]; however, no research has been published with respect to the characteristics of nasal isolates from healthcare staff. In the present study, we used molecular epidemiology and population analyses to reveal the differences in the genetic endowment and clonal lineages with the pathogenesis and resistance traits of isolates that were collected from community and hospital environments of the same ecological niche, time period, and geographical location in China.

## Materials and Methods

### Ethics Statement

The nasal screening of healthy volunteers and healthcare staff was approved by the ethics committee of Huashan Hospital, Shanghai Medical College, Fudan University, Shanghai, People's Republic of China. All subjects provided written informed consent before their inclusion in the study.

### Bacterial isolates

#### (a) Clinical isolates

120 no duplicate *S. epidermidis* isolates were collected randomly from 2010 to 2011 from inpatients with *S. epidermidis* infections in Shanghai Huashan hospital. This hospital is located in the center of Shanghai, China, and is a large (1300 bed) teaching hospital that handles ∼8000 admissions per day. The isolates were recovered from blood (38 isolates, 31.7%), catheters (39 isolates, 32.5%), and other sterile body fluids (43 isolates, 35.8%), including cerebrospinal fluid, articular cavity fluid, and pleural cavity fluid. *S. epidermidis* ATCC12228 and *S. epidermidis* RP62A, 2 reference strains of which their full genome sequences have been published, were also included in this study as controls. We also collected 104 no duplicate *Staphylococcus haemolyticus* isolates were also randomly collected from 2010 to 2011 from inpatients with *S. haemolyticus* infections in Shanghai Huashan hospital. Isolates recovered from sputum, urine, or wound samples were not included in this study as they were often considered to be contaminated.

#### (b) Community carriage of *S. epidermidis*

To contrast community carriage against the clinical isolates, 112 *S. epidermidis* isolates from nasal swabs were collected in 2011 as part of a population-based community prevalence study among 250 (44.8%) healthy volunteers in the Shanghai area of China. The healthy volunteers did not take any antibiotics and had no contact with the hospital in the 3 months prior to sampling and were considered to be community carriers of *S. epidermidis*.

#### (c) Nasal isolates from healthcare staff

During the same time period, 92 *S. epidermidis* nasal isolates were collected from 150 (61.3%) healthcare staff (doctors and nurses) in the same hospital. *S. epidermidis* was confirmed by classic microbiological methods: Gram stain and catalase and coagulase activity on rabbit plasma. The *S. epidermidis* strains were further identified by biochemical characterization using the Api-Staph test (bioMérieux, Lyon, France).

### Antimicrobial resistance profiles

Antibiograms of the isolates were generated using the disc diffusion method according to the 2011 guidelines of the Clinical and Laboratory Standards Institute (CLSI). The following antimicrobial agents were tested: gentamicin (10 mg), penicillin (10 IU), cefoxitin (30 mg), cefazolin (30 mg), vancomycin (30 mg), linezolid (30 mg), erythromycin (15 mg), clindamycin (2 mg), sulfamethoxazole (23.75 mg) plus trimethoprim (1.25 mg), fosfomycin (200 mg), rifampicin (5 mg), teicoplanin (30 mg), and levofloxacin (5 mg). In addition, the minimum inhibitory concentration of vancomycin was determined using the agar dilution method according to the guidelines of the CLSI.

### MLST and goeBURST algorithm

MLST was performed using the primer sequences and conditions described by Thomas *et al*. [21] for the PCR amplification of the 7 housekeeping genes *arcC*, *aroE*, *gtr*, *mutS*, *pyrR*, *tpiA*, and *yqiL*. The number of alleles and STs was determined by the well-characterized online MLST database (http://www.mlst.net). The goeBURST algorithm (http://goeBURST.phyloviz.net) was used to infer the evolutionary relatedness of the STs.

### Determination of SCC*mec* types

SCC*mec* types I–VI were determined by the combination of the type of *ccr* complex and the class of *mec* complex. The types of *ccr* and *mec* complexes were determined by a multiplex PCR approach using primers and conditions identical to those described by Kondo *et al*. [22]. SCC*mec* was defined as nontypeable when the *ccr* complex generated no other amplification products than *mecA* (286 bp) or the *mec* complex generated no amplification products in PCR amplification.

### Screening, typing, and sequencing ACME

The ACME-*arcA* and ACME-*opp3AB* genes were used as markers of the ACME-*arc* cluster and the ACME-*opp3* cluster, respectively. PCR screening was performed using the primers *arcA*-F (GAGCCAGAAGTACGCGAG) and *arcA*-R (CACGTAACTTGCTAGAACGAG) for ACME-*arcA* (671 bp); and *opp3*-F (GCAAATCTGTAAATGGTCTGTT) and *opp3*-R (GAAGATTGGCAGCACAAAGTG) for ACME-*opp3AB* (1183 bp). MRSA strain USA300-FPR3757 was used as a positive control for both PCRs. Amplicons were revealed by agarose gel electrophoresis and ethidium bromide staining. ACME was classified as type I (contains the ACME-*arcA* and ACME-*opp3AB* gene clusters), type II (carries only the ACME-*arcA* locus), and type III (carries only the ACME-*opp3AB* locus) [12].

ACME-*arcA* and ACME-*opp3AB* were sequenced in all positive strains using an ABI 3730 sequence analyzer. ACME-*arcA* and ACME-*opp3AB* identified in this study were compared with the reference sequences of ACME-*arcA* (USA300-FPR3757) and ACME-*opp3AB* (USA300-FPR3757). Phylogenic analysis of *arcA* and *opp3AB* was carried out using a neighbour-joining algorithm (no. of differences, distance estimation) with no outgroups by MEGA v5.0 software.

### *ica*- and *IS256*-specific PCR

Isolates were screened for the *ica* and *IS256* genes, which are related to biofilm formation, by PCR amplification using the primers described by Kozitskaya *et al.* [23]. The thermocycler conditions for the amplification of the *ica* and *IS256* genes also referred to those described by Kozitskaya *et al.* using *S. epidermidis* RP62A and ATCC12228 as controls.

### Semiquantitative biofilm assay

A semiquantitative biofilm assay was performed using 96-well tissue culture plates based on the method described by Wang *et al.* [24] with the following modifications. After the cells were fixed in Bouin's fixative for 1 h, the cells were washed gently 3 times in phosphate-buffered saline and then stained with 0.1% crystal violet solution. The stain was washed off gently under slowly running water and the plates were dried at room temperature. The absorbance of the stained biofilm was measured at 490 nm using a MicroELISA autoreader (Bio-Rad).

### Statistical analysis

The degree of genetic diversity was assessed by Simpson's index of diversity (SID), using a 95% CI. The χ2 test was used to analyze the quantitative variables. A P-value ≤ 0.05 was considered statistically significant.

## Results

### Diversity of the STs in *S. epidermidis* strains isolated from the community and hospital environments

A total of 44 STs were identified by MLST among the 324 *S. epidermidis* strains isolated from the community and hospital environments, which corresponds to a high level of genetic diversity (SID = 93.1%, 95% confidence interval [CI] 91.9–94.2%). Among the 120 clinical isolates, 25 STs were identified (SID = 84.3%, 95% CI 79.1–89.5%), with ST2 the most prevalent (42 isolates, 35.0%), followed by ST59 (18 isolates, 15.0%) (Table 1). Except for these 2 major STs, 47 isolates (39.2%) belonged to 10 minor STs: ST89 (8 isolates), ST262 (8 isolates), ST17 (6 isolates), ST5 (5 isolates), ST20 (4 isolates), ST130 (4 isolates), ST6 (3 isolates), ST173 (3 isolates), ST220 (3 isolates), and ST235 (3 isolates). The other 13 STs were singletons. In comparison with the clinical isolates, 19 STs were identified among 112 nasal colonization isolates from the community (SID = 91.4%, 95% CI 89.3–93.4%). The major STs were ST59 (21 isolates, 18.8%), ST17 (13 isolates, 11.6%), ST251 (12 isolates, 10.7%), and ST152 (11 isolates, 9.8%). The other 15 dispersed STs corresponded to 49.1% of the entire collection. The nasal colonization isolates recovered from healthcare staff were more clonal (SID = 94.0%, 95% CI 92.2–95.7%). We identified 27 STs among the 92 isolates, and only 36.0% of the isolates belonged to 3 major STs (ST20, ST59, and ST89, 11 isolates each).

**Table 1:** Molecular characterization of MRSE and MSSE strains isolated from community and hospital environments of Shanghai area of China during 2010 to 2011.

STs (No, %)	MRSE														MSSE							
	Total (%)	SCCmec type (%)					ACME allotypes (%)				IS256 (%)	icaA (%)	Biofilm (%)		Total (%)	ACME allotypes (%)				IS256 (%)	icaA (%)	Biofilm (%)
		III	IV	V	VI		I	II	III	total						I	II	III	total			
**Clinical isolates**																						
ST2 (42, 35.0)	40 (95.2)	39 (97.5)	1 (2.5)	0	0		5 (12.5)	11 (27.5)	0	16 (40.0)	34 (81.0)	30 (75.0)	17 (40.5)		2a (4.8)	0	0	0	0	1	1	0
ST59 (18, 15.0)	18 (100)	0	18 (100)	0	0		2 (11.1)	10 (55.6)	0	12 (66.7)	4 (22.2)	5 0	4 (22.2)		0	0	0	0	0	0	0	0
Other STsb (60, 50.0)	46 (76.7)	2 (4.3)	15 (32.6)	28 (60.9)	1 (2.2)		16 (34.8)	13 (28.3)	0	29 (63.0)	19 (41.3)	8 (13.0)	7 (15.2)		14 (23.3)	6 (42.9)	6 (42.9)	0	12 (85.7)	3 (21.4)	2 (14.3)	3 (21.4)
Total (120)	104 (86.7)	41 (39.4)	34 (32.7)	28 (26.9)	1 (1.0)		23 (22.1)	34 (32.7)	0	57 (54.8)	57 (54.8)	43 (41.3)	28 (26.9)		16 (13.3)	6 (37.5)	6 (37.5)	0	12 (75.0)	4 (25.0)	3 (18.8)	3 (18.8)
**Nasal isolates from healthcare staffs**																						
ST20 (11, 12.0)	5 (45.5)	0	2	3	0		4	0	0	4	2	2	1		6 (54.5)	2	3	1	6	0	0	0
ST59 (11, 12.0)	5 (45.5)	0	4	1	0		1	2	1	4	2	0	0		6 (54.5)	3	2	0	5	1	1	1
ST89 (11, 12.0)	5 (45.5)	0	2	3	0		4	1	0	5	3	0	0		6 (54.5)	2	3	1	6	1	0	0
Other STs (59, 64.0)	30 (50.8)	1 (3.3)	14 (46.7)	15 (50.0)	0		15 (50.0)	11 (36.7)	1 (3.3)	27 (90.0)	10 (33.3)	5 (16.7)	3 (10.0)		29 (49.2)	8 (27.6)	13 (44.8)	3 (10.3)	24 (82.6)	8 (27.6)	8 (27.6)	3 (10.3)
Total (92)	45 (48.9)	1 (2.2)	22 (48.9)	22 (48.9)	0		24 (53.3)	14 (31.1)	2 (4.4)	40 (88.9)	17 (37.8)	7 (15.6)	4 (8.9)		47 (51.1)	15 (31.9)	21 (44.7)	5 (10.6)	41 (87.2)	10 (21.3)	9 (19.1)	4 (8.5)
**Nasal isolates from community healthy people**																						
ST59 (21, 18.8)	11 (52.4)	0	11 (100.0)	0	0		1 (9.1)	7 (63.6)	0	8 (72.7)	0	0	1 (9.1)		10 (47.6)	1 (10.0)	8 (80.0)	0	9 (90.0)	0	0	0
ST17 (13, 11.6)	7 (53.8)	0	0	7	0		3	3	0	6	0	0	0		6 (46.2)	1	3	1	5	1	0	0
ST251 (12, 10.7)	4 (33.3)	0	0	4	0		3	1	0	4	0	1 (8.3)	0		8 (66.7)	1	5	0	6	0	1	0
ST152 (11, 9.8)	4 (36.4)	0	2	2	0		1	1	0	2	1 (9.1)	0	0		7 (63.6)	2	4	0	6	3	1	0
Other STs (55, 49.1)	17 (30.9)	0	6 (35.3)	11 (64.7)	0		5 (29.4)	8 (47.1)	0	13 (76.5)	0	4 (28.6)	3 (17.6)		38 (69.1)	11 (28.9)	20 (52.6)	0	31 (81.6)	2 (5.3)	5 (13.2)	3 (7.9)
Total (112)	43 (38.4)	0	19 (44.2)	24 (55.8)	0		13 (30.2)	20 (46.5)	0	33 (76.7)	1 (2.2)	5 (10.9)	4 (9.3)		69 (61.6)	16 (23.2)	40 (58.0)	1 (1.4)	57 (82.6)	6 (8.7)	7 (10.1)	3 (4.3)

a: ST with less than 10 isolates were not calculated “%”.

b: Other STs means the total number of the dispersed STs with less than 10 isolates.

**Figure 1:**
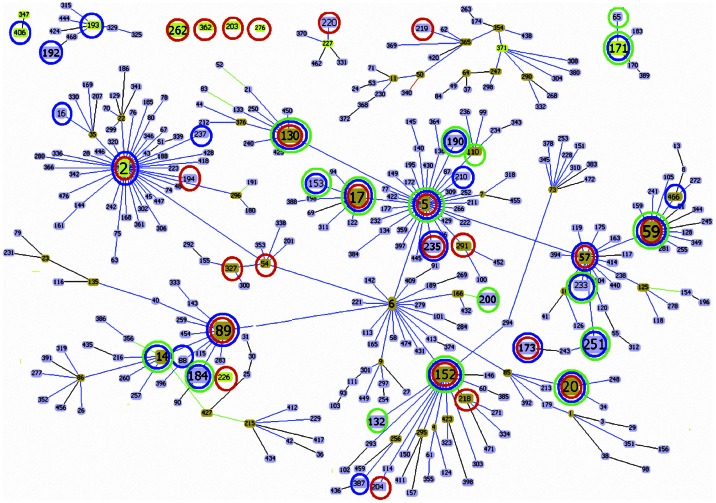
eBURST analysis of *S.* epidermidis using all STs available in the MLST database as of October 2012. ST nodes: Light green - Group founder; Dark green - Sub-group founder and Light blue - Common node. Other group founders which we did not find in this study were deleted. Red circle indicates STs isolated from clinical samples; Blue circle indicates STs isolated from Nasal samples of healthcare staffs; Green circle indicates STs isolated from Nasal samples of healthy people.

The goeBURST algorithm clustered all of the 44 STs isolated from the community and hospital environments into CC2 (297/324; 91.7%), 5 minor CCs (CC171, CC227, CC406, CC371, and CC193, 16/324, 4.9%), and 4 singletons (CC362, CC203, CC267, and CC262, 11/324, 3.4%) (Fig 1).When we compared the population structures of the isolates from patients, healthcare staff, and healthy individuals, we found that some STs were present in all of these populations; we named this group “genotype group 1.” All of the STs in genotype group 1 belonged to subgroup founders of CC2: CC2-II (CC2-II-5: ST5, ST59, ST17, and ST130; CC2-II-89: ST89; CC2-II-6: ST152; and CC2-II-85: ST20). The genotypes detected in patients and healthcare staff were defined as “genotype group 2,” which included ST2, ST57, ST173, and ST235. The majority of the isolates in genotype group 2 belonged to ST2 (75.4%, 43/57), and ST2 was the group founder of CC2. The genotypes identified in healthcare staff and healthy individuals were defined as “genotype group 3,” which included ST251, ST14, ST153, ST171, ST184, ST190, and ST233. The genotypes identified only in healthcare staff were defined as “genotype group 4” (ST54, ST194, ST203, ST204, ST218, ST219, ST220, ST226, ST262, ST267, ST291, ST327, and ST362), while those identified only in healthcare staff were defined as “genotype group 5” (ST16, ST88, ST192, ST193, ST210, ST237, ST387, ST406, and ST466). Finally, the genotypes identified only in healthy individuals were defined as “genotype group 6” (ST65, ST110, ST132, and ST200). ST6 was detected in patients and healthy individuals.

**Figure 2:**
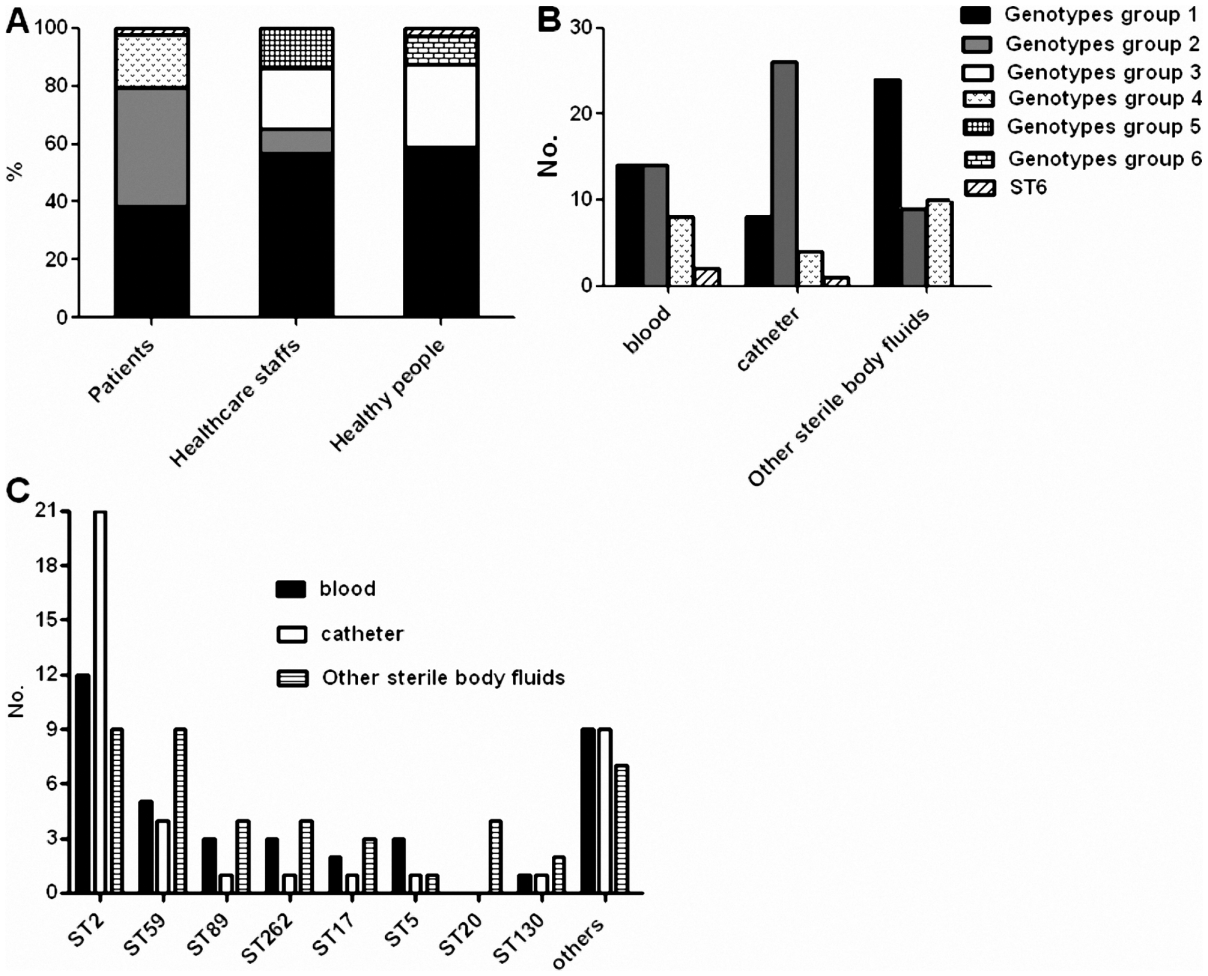
Distribution of different genotypes of *S.* epidermidis among different population structures and clinical samples. (A): The distribution of different genotypes groups among three population structures; (B): The distribution of genotypes groups isolated from patients among three clinical samples; (C): The distribution of different STs isolated from different clinical samples.

Fig 2A shows the distribution of the different genotypes groups among the 3 population groups. Only 40.8% of the isolates recovered from patients belonged to the same STs as those found in healthy individuals, but 77.2% of the isolates recovered from healthcare staff belonged to the same STs as those found in healthy individuals. The STs identified in the community were more frequently found in the healthcare staff than in the patients (P<0.001). However, several STs were only detected in the patients and healthcare staff, e.g., ST2, ST57, ST173, and ST235.

### Association of the genotypes and infection types caused by clinical *S. epidermidis* strains

Fig 2B shows the distribution of the different genotype groups among the 3 infection sites. Genotype group 1 was found more frequently in other sterile body fluids (52.2%, P<0.05). Most of the STs in genotype group 1, e.g., ST59, ST89, ST17, ST20, and ST130, were recovered more frequently from other sterile body fluid samples (Fig 2C). In contrast, genotype group 2 was more often recovered from catheter samples (P<0.001). The most prevalent genotype group 2 clones belonged to ST2 (85.7%, 42/49); Fig 2C shows that the predominant *S. epidermidis* clinical clone ST2 was mainly responsible for catheter-related infections.

### Frequency of MRSE, SCC*mec* typing, and antibiotic resistance of *S. epidermidis* strains isolated from the community and hospital environments

Of all the *S. epidermidis* isolates studied, 59.3% (192/324) were MRSE isolates. Among the 120 clinical isolates, 86.7% (104/120) were MRSE isolates (Table 1); 95.2% (40/42) of the *S. epidermidis* ST2 clinical clones were MRSE, and the predominant SCC*mec* type was ST2-SCC*mec*III (97.5%, 39/40). MRSE was observed in 100% (18/18) of the ST59 clones isolated from the clinical samples, and all ST59 clones carried SCC*mec*IV. The other 23 STs accounted for 50.0% of the clinical isolates; 60.9% of them carried SCC*mec*V. No SCC*mec*I or II was found in any of the *S. epidermidis* strains isolated from the community and hospital environments. Of the 92 *S. epidermidis* nasal isolates from healthcare staff, 48.9% (45/92) were MRSE; the majority of the MRSE isolates in this group carried SCC*mec*IV (48.9%, 22/45) and SCC*mec*V (48.9%, 22/45), while only 2.2% (1/45) of strains carried SCC*mec*III. The percentage of MRSE in nasal isolates from healthy individuals was 38.4% (43/112), similar to that of the healthcare staff (P>0.05), but significantly lower than that of the clinical isolates (P<0.001). MRSE from community carriage harbored SCC*mec*IV (44.2%, 19/43) and SCC*mec*V (55.8%, 24/43), which was also similar to the carriage of the healthcare staff (P>0.05), but was significantly different (P<0.001) from that of the clinical isolates.

A total of 324 *S. epidermidis* strains isolated from the community and hospital environments were analyzed for antimicrobial resistance. All isolates were susceptible to vancomycin, teicoplanin, and linezolid. Resistance to penicillin (90.4%, 293/324) was the most frequently observed. In addition to β-lactam antibiotics, the clinical strains showed significantly higher multiple antibiotic resistance profiles than the nasal isolates from healthcare staff and healthy individuals (Table 2). The predominant clinical clone ST2 was almost completely resistant to all antibiotics used in this study, except vancomycin, teicoplanin, and linezolid.

**Table 2:** Antimicrobial resistance profiles and molecular characterization of *S. epidermidis* isolates which could be detected in different populations.

populations	Total (%)	MRSA (%)	Other antibiotic resistance (%)								ACME allotypes (%)				*IS256* (%)	*icaA* (%)	Biofilm (%)
			GM	P	E	CLI	SXT	FOS	LEV		I	II	III	total			
**Genotypes group 1^a^:**																	
Patients	46 (38.3)	40 (87.0)	13 (28.3)	43 (93.8)	38 (82.6)	21 (45.7)	27 (58.7)	10 (21.7)	26 (56.5)		13 (28.3)	21 (45.7)	0	34 (73.9)	12 (26.1)	10 (21.7)	11 (23.9)
Healthcare staffs	52 (56.5)	27 (51.9)	11 (21.2)	47 (90.4)	30 (57.7)	13 (25.0)	21 (40.4)	7 (13.5)	18 (34.6)		23 (44.2)	19 (36.5)	4 (7.7)	46 (88.5)	14 (26.9)	7 (13.5)	4 (7.7)
Healthy people	66 (58.9)	32 (48.5)	7 (10.6)	59 (89.4)	33 (50.0)	14 (21.2)	38 (57.6)	0	4 (6.1)		15 (22.7)	36 (54.5)	1 (1.5)	52 (78.8)	6 (9.1)	4 (6.1)	4 (6.1)
**Genotypes group 2:**																	
Patients	49 (40.8)	46 (93.9)	42 (85.7)	49 (100.0)	43 (87.8)	39 (79.6)	45 (91.8)	39 (79.6)	45 (91.8)		11 (22.4)	12 (24.5)	0	23 (46.9)	41 (83.7)	34 (69.4)	20 (40.8)
Healthcare staffs	8^b^ (8.7)	4	0	8	2	1	5	0	1		5	3	0	8	2	2	1
**Genotypes group 3:**																	
Healthcare staffs	19 (20.7)	10 (52.6)	7 (36.8)	17 (89.5)	7 (36.8)	0	8 (42.1)	3 (15.8)	8 (42.1)		7 (36.8)	8 (42.1)	2 (10.5)	17 (89.5)	8 (42.1)	6 (31.6)	3 (15.8)
Healthy people	32 (28.6)	8 (25.0)	2 (6.3)	28 (87.5)	10 (31.3)	4 (12.5)	13 (40.6)	1 (3.1)	1 (3.1)		7 (21.9)	20 (62.5)	0	27 (84.4)	1 (3.1)	6 (18.8)	2 (6.3)
**Genotypes group 4:**																	
Patients	22 (18.3)	15 (68.2)	4 (18.2)	20 (90.9)	18 (81.8)	4 (18.2)	9 (40.9)	1 (4.5)	7 (31.2)		5 (22.7)	5 (22.7)	0	10 (45.5)	6 (27.3)	2 (9.1)	0
**Genotypes group 5:**																	
Healthcare staffs	13 (14.1)	4 (30.8)	1 (7.7)	9 (69.2)	8 (61.5)	4 (30.8)	2 (15.4)	0	5 (38.5)		4 (30.8	5 (38.5)	1 (7.7)	10 (76.9)	3 (23.1)	1 (7.7)	0
**Genotypes group 6:**																	
Healthy people	11 (9.8)	3 (27.3)	0	9 (81.8)	2 (18.2)	2 (18.2)	0	1 (9.1)	1 (9.1)		6 (54.5)	3 (27.3)	0	9 (81.8)	0	1 (9.1)	0
**ST6**																	
Patients	3	3	2	3	2	2	0	2	3		0	2	0	2	2	0	0
Healthy people	3	0	0	1	1	1	0	0	0		1	1	0	2	0	1	1

a: genotypes group 1 included STs could be detected in all of three populations; genotypes group 2 included STs could be detected in both patients and healthcare staffs; genotypes group 3 included STs could be detected in both healthcare staffs and healthy people; genotypes group 4 included STs could only be detected in patients; genotypes group 5 included STs could only be detected in healthcare staffs; genotypes group 6 included STs could only be detected in healthy people.

b: ST with less than 10 isolates were not calculated “%”.

### Prevalence of ACME in *S. epidermidis* strains isolated from the community and hospital environments

A high prevalence (74.1%, 240/324) of ACME was found in *S. epidermidis* strains in this study; 67.7% (130/192) of MRSE isolates and 83.3% (110/132) of MSSE isolates carried ACME, with MSSE harboring significantly more ACME than MRSE (P<0.05). The prevalence of ACME in clinical isolates and MRSE-positive healthcare staff and healthy individuals was 54.8%, 88.9%, and 76.7%, respectively. The distribution of ACME in clinical MRSE isolates was significantly lower than in nasal isolates from healthy individuals and healthcare staff (P<0.001). Only 40.0% of the predominant clinical MRSE clone ST2 carried ACME. Among the 130 ACME-positive MRSE isolates, 46.1% (60 isolates), 52.3% (68 isolates), and 1.5% (2 isolates) carried ACME type I, II, and III, respectively. The prevalence of ACME in MSSE-positive clinical isolates, healthcare staff, and healthy individuals was 75.0%, 87.2%, and 82.6%, respectively; no significant difference was found among the 3 groups (P>0.05). We found that 33.6% (37 isolates), 60.9% (67 isolates), and 5.5% (6 isolates) of the ACME-positive MSSE isolates carried ACME type I, II, and III, respectively; there was no significant difference in the distribution of these 3 ACME allotypes in MRSE and MSSE isolates (P>0.05). In this study, 19.2% (20/104) of *S. haemolyticus* clinical isolates carried ACME, and all of the ACME-positive *S. haemolyticus* isolates carried ACME type II.

The ACME-*arcA* gene from the 99-positive MRSE strains that belonged to genotype group 1 together with the ACME-positive *S. haemolyticus* clinical isolates could be split into 4 distinct allotypes (Figure S1). When compared with its counterpart in USA300-FPR3757, most of the ACME-*arcA* genes (92.9%, 92/99) harbored by these MRSE strains displayed 100% nucleotide (nt) identity.Eleven *S. haemolyticus* clinical isolates carried the other 2 distinct allotypes of the ACME-*arcA* gene, and 6 of them showing 100% nt identity with the MSSE strain ATCC12228.

The ACME-*opp3AB* from the 42 MRSE-positive strains belonging to genotype group I were split into 9 distinct allotypes (Figure S2). Compared with USA300-FPR3757, only four strains harboring an ACME-*opp3AB* gene displayed 100% nt identity, which also carried the same ACME-*arcA* gene as USA300-FPR3757. The remaining 38 strains carried ACME-*opp3AB* allotypes with 1–30 nt substitutions when compared with their counterpart in USA300-FPR3757, showing 97–99% identity.

### Biofilm formation and the presence of related genes in *S. epidermidis* strains isolated from the community and hospital environments

A total of 54.8% (57/104) and 41.3% (43/104) of the clinical MRSE isolates carried the *IS256* and *icaA* genes, whereas 25.0% (4/16) and 18.8% (3/16) of the clinical MSSE isolates harbored these genes (P<0.05). The *IS256* and *icaA* genes were detected in 16.7% (34/204) and 13.7% (28/204) of nasal isolates, which was significantly lower than in the clinical isolates (P<0.05). Biofilm production was observed in 26.9% (28/104) of the clinical MRSE isolates and 18.8% (3/16) of the clinical MSSE isolates. Only 8.7% (8/92) of the nasal isolates from healthcare staff and 6.3% (7/112) from healthy individuals were biofilm-producing isolates. Biofilm formation in the nasal isolates was significantly lower than in the clinical isolates (P<0.001). Biofilm production was observed in 41.1% (39/95) of the *IS256*-positive isolates and 56.8% (42/74) of the *icaA*-positive isolates. Biofilm formation was observed significantly more in isolates carrying an *icaA* gene than in those with an *IS256* gene (P<0.05). The biofilm-related genes *IS256* and *icaA* were detected in the majority (81.0% and 75.0%) of the predominant clinical MRSE clone ST2 isolates, and 40.5% (17/40) of them were biofilm positive.

## Discussion

In the present study, we found a high nasal colonization rate with *S. epidermidis* (44.8%) in the healthy individuals in the Shanghai area of China, with an MRSE colonization rate of 17.2%. This result is in line with other studies on healthy populations that observed a similar MRSE colonization rate, e.g., 20% in healthy Brazilian individuals [25] and 19.3% in healthy Japanese children [18]. The nasal colonization rates of *S. epidermidis* and MRSE in the healthcare personnel in a Shanghai hospital were 61.3% and 30.0%, respectively, which were much higher than that in the healthy population, and some of these *S. epidermidis* clones could only be detected in patients and healthcare personnel. This result supports the concerns that medical and healthcare staff could act as a reservoir for pathogenic bacteria.

Molecular characterization of the 324 *S. epidermidis* strains isolated from the community and hospital environments of the same geographical region and comparable time periods by MLST identified 44 STs, indicating a high level of genetic diversity at slowly evolving loci. A high level of diversity within *S. epidermidis* was also observed in other studies [14], [26]. It was supposed that this genetic diversity might be caused by the need of the isolates to adapt to the different environments in the hospital and community settings [3]. The comparison of *S. epidermidis* strains isolated from the community and patients showed that both populations had a high level of genetic diversity, but the community population was even more diverse than the infection-related population. Only 40.8% of isolates recovered from the patients belonged to the same STs as those found in the healthy individuals. This indicates that certain *S. epidermidis* clones probably have particular genetic features that may be related to their capacity to survive in different environments. In spite of the observation that 44 different STs were found in all *S. epidermidis* strains analyzed, the great majority of the isolates from either the community or hospital belonged to a single clonal complex, i.e., CC2. This clonal complex has been described previously as the most prevalent one in the nosocomial population of *S. epidermidis* [3], being characterized by a high level of genetic diversity, an increased recombination/mutation rate, and a high number of SCC*mec* elements. In our study, most of the STs that could only be detected in the community also belonged to CC2, suggesting that CC2 lineages have used recombination/mutation to adapt to environments with distinct characteristics.

In this study, MRSE ST2-SCC*mec*III was the predominant clone in the clinical isolates. ST2 is usually the most prevalent ST in previous epidemiological studies among *S. epidermidis* causing hospital-acquired infections worldwide [16], [27]–[29]. The high rate of *S. epidermidis* ST2-caused infections poses the question as to why this clone has established itself so successfully as an opportunistic pathogen in the healthcare setting. According to the current study, 2 main factors contribute to the pathogenesis of *S. epidermidis* ST2-related infections. (a) High multiple antibiotic resistance profile of ST2: 95.2% of the predominant clinical ST2 clones were MRSE, and it was almost completely resistant to all of the antibiotics tested in this study, except vancomycin, teicoplanin, and linezolid. It was supposed that ST2 might be more prone to acquire, maintain, and express resistance genes. The ability of this lineage to acquire resistance genes renders it highly adapted to the nosocomial environment. Most of the MRSE ST2 isolates carried the SCC*mec*III cassette, except for 1 isolate that harbored SCC*mec*IV. Most of the other clinical isolates carried SCC*mec*IV or SCC*mec*V cassettes. The majority of MRSE isolates from medical staff and healthy individuals also carried an SCC*mec*IV or SCC*mec*V cassette. SCC*mec*IV is reportedly the most frequently acquired SCC*mec* by *S. epidermidis*, which is in accordance with the enhanced mobility of this type of SCC*mec* observed in *S. aureus* [3]. It is unclear why the SCC*mec*III cassette was the particular genetic feature of the ST2 isolates in this Chinese hospital. One possible reason is that specific physiological conditions during infection and stresses imposed by the hospital environment can promote SCC*mec*III acquisition/dissemination in the ST2 strains. (b) Biofilm formation in multiple antibiotic resistance clone ST2: *S. epidermidis* developed from a commensal bacterium into a pathogen by its ability to form thick, multilayered biofilms on inert surfaces [30], [31]. Bacteria organized in biofilms are more resistant to antibiotics and can cope much better with unfavorable external conditions than their planktonic counterparts. In this study, biofilm formation was much lower in the nasal isolates than in the clinical isolates. The biofilm-related genes *IS256* and *icaA* were detected in the majority of the predominant clinical MRSE ST2 clones, and 40.5% of them were biofilm positive. From our data, the presence of the *icaA* gene was more related to biofilm production than the *IS256* gene; however, in addition to the modulation of biofilm formation, IS*256* is also involved in the transcriptional regulation of resistance genes and the inactivation of global gene regulators [32], [33]. Therefore, the formation of a biofilm represents a benefit for the predominant MRSE ST2-SCC*mec*III clone, enabling it to colonize the inert surfaces of medical devices and to resist a wide range of external conditions, thus causing device-related infections. No ST2 isolate was found in the community setting in the present study; it is likely that ST2 is highly adapted to the hospital environment and differs from commensal *S. epidermidis* in the community. It is possible that patients who are admitted to hospital are soon colonized by these biofilm-forming, multiresistant *S. epidermidis* isolates and that this newly acquired endogenous microflora might represent the origin for a later infection.

ACME is a genomic island in *Staphylococci* that was first discovered in the community-associated MRSA (CA-MRSA) USA300 strain and was believed to be a survival factor for this species [34]. ACME appears to have been transferred from *S. epidermidis* into *S. aureus* [35]. In this study, we found a high prevalence (74.1%) of ACME in *S. epidermidis*. This result is in agreement with the report of Diep *et al.* 10] in which 67% of *S. epidermidis* nasal isolates were ACME positive, and the report of Miragaia *et al.* [35] in which 51% of *S. epidermidis* isolates from a widespread geographical origin were ACME positive. A recent study showed that 48% of clinical methicillin-resistant *S. haemolyticus* isolates carried ACME [36]; however, we found that ACME was only carried by 19.2% of clinical *S. haemolyticus* isolates. *S. haemolyticus* isolates harbored an ACME-*arcA* gene with high identity to *S. epidermidis* ATCC12228, suggesting that *S. haemolyticus* obtained ACME from *S. epidermidis*.

In this study, ACME types I and II, both containing an ACME-*arc* cluster, accounted for 96.7% (232/240) of the ACME-positive *S. epidermidis* isolates. ACME-*arcA* exhibited highly conserved sequences, and most of the strain types that could be detected in the community and hospital environments carried the ACME-*arcA* gene with high identity to USA300-FPR3757. However, the prevalence of ACME-*opp3* in *S. epidermidis* isolates was relatively low when compared with ACME-*arc* (32.4% versus 71.6%, respectively). ACME-*opp3AB* displayed a wide diversity of sequences in our study, contrasting with the highly conserved nature of ACME-*arcA*. This result is consistent with the findings of Barbier *et al*.in MRSE [12], and suggests that ACME-*opp3* may be less crucial than ACME-*arc* in terms of fitness benefit for carriage strains of *S. epidermidis*.

Studies in *S. aureus* show that ACME is often integrated in the bacterial chromosome adjacent to a SCC*mec*IV element [10], [11], [37]. However, in our study, MSSE isolates harbored more ACME (83.3%) than MRSE isolates (67.7%), which is in line with the study of Granslo *et al.* [38], in which a significantly lower rate of methicillin and gentamicin resistance was observed between ACME-positive versus ACME-negative *S. epidermidis* isolates. No association between ACME and SCC*mec* type IV in *S. epidermidis* was found in our study, which is also similar to the results of Granslo *et al.* [38]. It is suggested that the recombinases used to integrate ACME and SCC*mec* into the bacterial chromosome are able to integrate these 2 genetic elements independently, and different selection mechanisms may apply for *S. aureus* and *S. epidermidis*. In *S. aureus*, ACME may contribute to enhanced pathogenicity. However, in this study, the distribution of ACME in the clinical *S. epidermidis* isolates (54.8%) was significantly lower than in the nasal isolates from healthy individuals (88.9%) and medical staff (76.7%), with only 40.0% of biofilm-forming, multiresistant clinical ST2 clones carried ACME. We found an association between the low level of ACME and invasive *S. epidermidis* infections in this study. Thus, we speculate that ACME in *S. epidermidis* may be considered as an indicator of benign, colonizing isolates. The avoidance of carrying large genetic elements such as ACME, which is not very necessary for the virulence of *S. epidermidis*, may theoretically increase the ability of strains to grow and survive in the hospital environment.

In conclusion, this study provides information on the epidemiology and molecular characteristics of *S. epidermidis* strains isolated from community and hospital environments in the same geographical region and comparable time periods in China. The *S. epidermidis* studied showed a high level of diversity, with some STs adapted more to the community environment with a low-level of antibiotic pressure, while others were adapted more to the selective environment of a hospital. Only 40.8% of isolates recovered from patients belonged to the same STs as those found in healthy individuals. In addition, healthcare staff might act as a reservoir for pathogenic bacteria. CC2 comprised a large number of the STs from either the community or hospital environment, suggesting its strong ability (e.g., resistant genes obtaining, biofilm forming) to adapt to both low- and high-level antibiotic environments, and it might continue to be the dominant clonal complex over the next few years. In contrast to the observation that biofilm formation and multiple antibiotic resistance showed a close association with invasive infections caused by *S. epidermidis*, ACME in *S. epidermidis* is more likely to be an indicator for colonization within a host, rather than a virulence factor.

## Supporting Information

Figure S1 **Neighbour-joining tree based on the comparison of 671 bp internal regions of ACME-** ***arcA*** **genes from 99 MRSE isolates, MSSE strain ATCC12228 and MRSA strain USA300-FPR3757.**
(TIF)

Figure S2 **Neighbour-joining tree based on the comparison of 1183 bp internal regions of ACME-** ***opp3AB*** **genes from 42 MRSE strains and MRSA strain USA300-FPR3757.** IR stands for infection related *S. epidermidis* isolates; NH stands for *S. epidermidis* isolates from nasal of healthcare staff; NC stands for *S. epidermidis* isolates from community health people. (I), (II), and (III) stands for type I ACME, type II ACME, and type III ACME, respectively.
(TIF)
